# SIX MONTHS OF GEROFIT RESULTS IN MOBILITY IMPROVEMENTS IN OLDER VETERANS REGARDLESS OF BMI CLASSIFICATION

**DOI:** 10.1093/geroni/igz038.134

**Published:** 2019-11-08

**Authors:** Odessa Addison, Jamie Giffuni, Monica Serra, Leslie Katzel

**Affiliations:** 1 Baltimore GRECC VAMHCS, Baltimore, Maryland, United States; 2 Geriatric Research, Education, and clinical center, baltimore, Maryland, United States; 3 Atlanta VA, Atlanta, Georgia, United States

## Abstract

Older Veterans represent a unique population at high risk for mobility limitations. They are also more likely to be overweight or obese when compared to the general population. We sought to compare changes in mobility function across the obesity spectrum in older Gerofit participants at six different sites. Two-hundred and seventy Veterans (mean age: 74 years) completed six-months of Gerofit participation and mobility assessments at baseline, three and six months. Our assessments included gait-speed, six-minute walk distance, 30-second chair stands, and the eight foot up and go. When comparing weight groups, we found no significant interaction of weight and time, however we found clinically significant (P<0.02) improvements of 7-20% across all mobility measures. Six-months of Gerofit participation appears to be one way to improve mobility function in older Veterans across the weight spectrum.

